# Supplementary material for: Self-other generalisation shapes social interaction and is disrupted in borderline personality disorder
Source: eLife. 2025 Jul 14;14:RP104008. doi: 10.7554/eLife.104008 (PMC12259023; doi:10.7554/eLife.104008)
Supplement: Supplementary file 1. — Option pair rewards for each phase and their corresponding ‘type’. Within phase, the order of trials was randomised. P=Prosocial, I=Individualistic, C=Competitive. S1=reward to self for option 1. S2=reward to self for option 2. O1=reward to other for option 1. O2=reward to other for option 2. [file elife-104008-supp1.docx]

| **S1** | **O1** | **S2** | **O2** | **Self-Disparity** | **Other Disparity** | **Type** |
| --- | --- | --- | --- | --- | --- | --- |
| 10 | 6 | 8 | 2 | 2 | 4 | I-C |
| 7 | 7 | 10 | 7 | 3 | 0 | P-I |
| 7 | 1 | 8 | 5 | 1 | 4 | C-I |
| 10 | 5 | 10 | 10 | 0 | 5 | C-P |
| 12 | 9 | 9 | 9 | 3 | 0 | I-P |
| 10 | 5 | 8 | 1 | 2 | 4 | I-C |
| 6 | 2 | 8 | 6 | 2 | 4 | C-I |
| 8 | 2 | 9 | 6 | 1 | 4 | C-I |
| 5 | 5 | 5 | 1 | 0 | 4 | P-C |
| 7 | 7 | 7 | 2 | 0 | 5 | P-C |
| 12 | 8 | 8 | 8 | 4 | 0 | I-P |
| 8 | 8 | 8 | 2 | 0 | 6 | P-C |
| 9 | 5 | 7 | 1 | 2 | 4 | I-C |
| 6 | 6 | 8 | 6 | 2 | 0 | P-I |
| 6 | 1 | 7 | 5 | 1 | 4 | C-I |
| 12 | 6 | 10 | 2 | 2 | 4 | I-C |
| 7 | 7 | 7 | 1 | 0 | 6 | P-C |
| 10 | 6 | 6 | 6 | 4 | 0 | I-P |
| 4 | 4 | 8 | 4 | 4 | 0 | P-I |
| 11 | 6 | 9 | 2 | 2 | 4 | I-C |
| 5 | 1 | 7 | 5 | 2 | 4 | C-I |
| 8 | 5 | 8 | 8 | 0 | 3 | C-P |
| 6 | 6 | 10 | 6 | 4 | 0 | P-I |
| 6 | 3 | 6 | 6 | 0 | 3 | C-P |
| 5 | 5 | 8 | 5 | 3 | 0 | P-I |
| 11 | 5 | 9 | 1 | 2 | 4 | I-C |
| 9 | 5 | 9 | 9 | 0 | 4 | C-P |
| 7 | 3 | 7 | 7 | 0 | 4 | C-P |
| 8 | 3 | 8 | 8 | 0 | 5 | C-P |
| 12 | 10 | 10 | 10 | 2 | 0 | I-P |
| 10 | 8 | 8 | 8 | 2 | 0 | I-P |
| 6 | 6 | 6 | 1 | 0 | 5 | P-C |
| 6 | 6 | 6 | 2 | 0 | 4 | P-C |
| 7 | 2 | 8 | 6 | 1 | 4 | C-I |
| 10 | 7 | 7 | 7 | 3 | 0 | I-P |
| 8 | 8 | 10 | 8 | 2 | 0 | P-I |
