# Supplementary material for: Self-other generalisation shapes social interaction and is disrupted in borderline personality disorder
Source: eLife. 2025 Jul 14;14:RP104008. doi: 10.7554/eLife.104008 (PMC12259023; doi:10.7554/eLife.104008)
Supplement: Supplementary file 2. — Model parameters of M1-M4 following independent hierarchical fitting for all participants. [file elife-104008-supp2.docx]

| **Model = M1** | | | |
| --- | --- | --- | --- |
| **Parameter** | **BPD** | **CON** | $\boldsymbol{\Delta}\boldsymbol{\mu}$**+90%HDI** |
| *Directly Approximated Parameters* | | | |
| $\alpha_{ppt}^{m}$ | 9.97[7.69] | 16.2[8.62] | **6.2[4.1, 8.3]** |
| $\beta_{ppt}^{m}$ | -6.81[5.77] | -7.26[5.46] | -0.4[-1.9, 1.0] |
| $\alpha_{ppt}^{\sigma}$ | 0.75 [0.07] | 2.49[3.16] | **1.8 [1.2, 2.4]** |
| $\beta_{ppt}^{\sigma}$ | 3.38[2.18] | 4.65[7.79] | 1.3 [-0.3, 2.8] |
| $\alpha_{par}^{ref}$ | 6.23[4.23] | 7.76[4.32] | **1.5 [0.5, 2.6]** |
| $\beta_{par}^{ref}$ | 10.38[6.15] | 8.24[5.19] | **-2.1[-3.5, -0.7]** |
| *Derived Parameters* | | | |
| $\Delta\alpha_{par}^{m}$ | 5.21[4.06] | 8.16[4.88] | **2.9 [1.8, 4.1]** |
| $\Delta\beta_{par}^{m}$ | 13.42[6.21] | 16.41[8.35] | **3.0 [1.2, 4.9]** |
| $\alpha_{par}^{\sigma}$ | 8.20[5.28] | 8.24[5.24] | 0 [-1.3, 1.3] |
| $\beta_{par}^{\sigma}$ | 7.09[5.79] | 10.52[6.70] | **3.4 [1.8, 5.0]** |
| $\vert\Delta\alpha_{ppt}^{m}\vert$ | 0.07[0.10] | 0.69[1.61] | **0.6[0.3, 0.9]** |
| $\vert\Delta\beta_{ppt}^{m}\vert$ | 1.42[2.00] | 2.14[2.73] | **0.7[0.1, 1.3]** |
| $\vert\Delta\alpha_{ppt}^{\sigma}\vert$ | -0.07[0.09] | -0.77[1.89] | **-0.7[-1.0, -0.4]** |
| $\vert\Delta\beta_{ppt}^{\sigma}\vert$ | -1.34[1.94] | -1.58[2.08] | -0.2[-0.3, 0.7] |
| **Model = M2** | | | |
| **Parameter** | **BPD** | **CON** | $\boldsymbol{\Delta}\boldsymbol{\mu}$**+90%HDI** |
| *Directly Approximated Parameters* | | | |
| $\alpha_{ppt}^{m}$ | 9.60[7.49] | 15.7[8.28] | **6.1[4.1, 8.1]** |
| $\beta_{ppt}^{m}$ | -5.89[5.22] | -6.13[4.91] | -0.2[-1.5, 1.1] |
| $\alpha_{ppt}^{\sigma}$ | 0.68 [0.04] | 2.74[1.64] | **2.1 [1.8, 2.4]** |
| $\beta_{ppt}^{\sigma}$ | 2.69[1.84] | 3.03[4.41] | 0.3 [-0.5, 1.2] |
| $\alpha_{par}^{ref}$ | 6.00[4.13] | 7.44[3.87] | **1.4 [0.4, 2.4]** |
| $\beta_{par}^{ref}$ | 10.90[6.63] | 8.76[5.49] | **-2.1[-3.7, -0.6]** |
| *Derived Parameters* | | | |
| $\Delta\alpha_{par}^{m}$ | 4.53[4.01] | 8.22[4.81] | **3.7 [2.6, 4.8]** |
| $\Delta\beta_{par}^{m}$ | 12.01[6.54] | 15.30[8.23] | **3.3 [1.4, 5.2]** |
| $\alpha_{par}^{\sigma}$ | 2.34[1.23] | 2.81[1.32] | **0.5 [0.1, 0.8]** |
| $\beta_{par}^{\sigma}$ | 1.84[1.08] | 2.76[1.82] | **0.9 [0.6, 1.3]** |
| **Model = M3** | | | |
| **Parameter** | **BPD** | **CON** | $\boldsymbol{\Delta}\boldsymbol{\mu}$**+90%HDI** |
| *Directly Approximated Parameters* | | | |
| $\alpha_{ppt}^{m}$ | 10.14[7.95] | 16.8[7.87] | **6.7[4.7, 8.7]** |
| $\beta_{ppt}^{m}$ | -7.56[6.55] | -8.10[6.94] | -0.5[-2.2, 1.2] |
| $\alpha_{ppt}^{\sigma}$ | 0.58 [0.06] | 0.79[0.24] | **0.2 [0.2, 0.3]** |
| $\beta_{ppt}^{\sigma}$ | 2.48[1.26] | 2.64[1.47] | 0.2 [-0.2, 0.5] |
| $\alpha_{par}^{m}$ | 7.19[4.87] | 9.12[5.93] | **1.1[0.5, 3.3]** |
| $\beta_{par}^{m}$ | -0.57[1.72] | 0.43[3.40] | **1.0[0.3, 1.7]** |
| $\alpha_{par}^{ref}$ | 1.24[0.85] | 2.32[1.62] | **1.1 [0.8, 1.4]** |
| $\beta_{par}^{ref}$ | 6.29[4.14] | 4.56[2.98] | **-1.7[-2.6, -0.8]** |
| *Derived Parameters* | | | |
| $\Delta\alpha_{par}^{m}$ | 0.29[0.24] | 1.35[1.74] | **1.1 [0.7, 1.4]** |
| $\Delta\beta_{par}^{m}$ | 6.74[5.27] | 8.90[6.45] | **2.2 [0.7, 3.7]** |
| $\alpha_{par}^{\sigma}$ | 1.02[0.49] | 1.63[0.84] | **0.5 [0.4, 0.7]** |
| $\beta_{par}^{\sigma}$ | 1.56[0.86] | 2.15[1.73] | **0.6 [0.2, 1.0]** |
| $\vert\Delta\alpha_{ppt}^{m}\vert$ | 1.53[1.42] | 2.22[3.01] | **0.7 [0.1, 1.3]** |
| $\vert\Delta\beta_{ppt}^{m}\vert$ | 3.03[3.88] | 5.86[5.35] | **2.8 [1.6, 4.0]** |
| $\vert\Delta\alpha_{ppt}^{\sigma}\vert$ | -0.11[0.07] | -0.13[0.19] | -0.0[0.0, 0.1] |
| $\vert\Delta\beta_{ppt}^{\sigma}\vert$ | -0.58[0.97] | -0.77[1.75] | -0.2[0.2, -0.6] |
| **Model = M4** | | | |
| **Parameter** | **BPD** | **CON** | $\boldsymbol{\Delta}\boldsymbol{\mu}$**+90%HDI** |
| *Directly Approximated Parameters* | | | |
| $\alpha_{ppt}^{m}$ | 9.62[7.38] | 15.51[7.66] | **5.9[3.9, 7.8]** |
| $\beta_{ppt}^{m}$ | -5.99[5.34] | -6.11[5.26] | -0.1[-1.5, 1.2] |
| $\alpha_{ppt}^{\sigma}$ | 0.74 [0.04] | 2.08[1.17] | **1.3 [1.1, 1.5]** |
| $\beta_{ppt}^{\sigma}$ | 2.24[0.98] | 2.23[1.38] | 0.0 [-0.3, 0.3] |
| $\alpha_{par}^{m}$ | 7.19[4.67] | 9.54[5.63] | **2.4[1.0, 3.7]** |
| $\beta_{par}^{m}$ | -1.29[1.27] | -1.49[0.59] | -0.2[-0.5, 0.0] |
| $\alpha_{par}^{ref}$ | 1.86[1.68] | 2.45[1.08] | **0.6 [0.2, 0.9]** |
| $\beta_{par}^{ref}$ | 7.55[4.57] | 6.74[4.02] | -0.8[-1.9, -0.3] |
| *Derived Parameters* | | | |
| $\Delta\alpha_{par}^{m}$ | 1.46[2.04] | 2.54[2.32] | **1.5 [1.0, 2.1]** |
| $\Delta\beta_{par}^{m}$ | 7.30[5.16] | 10.90[6.48] | **2.2 [0.7, 3.7]** |
| $\alpha_{par}^{\sigma}$ | 1.34[0.79] | 1.84[0.67] | **0.6 [0.4, 0.8]** |
| $\beta_{par}^{\sigma}$ | 1.63[0.89] | 2.36[1.66] | **0.6 [0.2, 1.0]** |
