# Supplementary material for: Self-other generalisation shapes social interaction and is disrupted in borderline personality disorder
Source: eLife. 2025 Jul 14;14:RP104008. doi: 10.7554/eLife.104008 (PMC12259023; doi:10.7554/eLife.104008)
Supplement: Supplementary file 3. — Random-effect linear relationships between DKL, trial, group, and preferences type for each model (M1-M4) following independent hierarchical fitting for all participants. Estimates are the scaled change in DKL as a result of each fixed effect. ID was used as a random variable to control for within-subject effects. Group effects (CON vs borderline personality disorder [BPD]) were analysed for the DKL within each preference type. [file elife-104008-supp3.docx]

| **M1** | | | | |
| --- | --- | --- | --- | --- |
|  | **Est.** | **se** | **t** | **p** |
| **Type** $[\beta vs.\alpha]$ | 0.21 | 0.019 | 10.99 | <2e-16 |
| **Trial** | -0.015 | 0.0001 | -23.87 | <2e-16 |
| **Type x Trial** | -0.016 | 0.001 | -13.36 | <2e-16 |
| **Group** $\left[ {D_{KL}(\alpha}_{par}^{m} \right)]$ | 0.083 | 0.030 | 2.78 | 0.006 |
| **Group** $\left[ {D_{KL}(\beta}_{par}^{m} \right)]$ | -0.064 | 0.028 | -2.31 | 0.023 |
| **M2** | | | | |
|  | **Est.** | **se** | **t** | **p** |
| **Type** $\boldsymbol{\beta vs.\alpha}$ | 0.21 | 0.019 | 10.92 | <2e-16 |
| **Trial** | -0.014 | 0.0001 | -22.70 | <2e-16 |
| **Type x Trial** | -0.016 | 0.0001 | -12.88 | <2e-16 |
| **Group** $\left[ {D_{KL}(\alpha}_{par}^{m} \right)]$ | 0.090 | 0.030 | 3.02 | 0.003 |
| **Group** $\left[ {D_{KL}(\beta}_{par}^{m} \right)]$ | -0.064 | 0.030 | -2.16 | 0.033 |
| **M3** | | | | |
|  | **Est.** | **se** | **t** | **p** |
| **Type** $\boldsymbol{\beta vs.\alpha}$ | 0.27 | 0.019 | 14.47 | <2e-16 |
| **Trial** | -0.0076 | 0.001 | -11.52 | <2e-16 |
| **Type x Trial** | -0.014 | 0.001 | -11.49 | <2e-16 |
| **Group** $\left[ {D_{KL}(\alpha}_{par}^{m} \right)]$ | 0.254 | 0.051 | 4.92 | 3.37e-06 |
| **Group** $\left[ {D_{KL}(\beta}_{par}^{m} \right)]$ | -0.360 | 0.050 | -7.71 | 7.56e-12 |
| **M4** | | | | |
|  | **Est.** | **se** | **t** | **p** |
| **Type** $\boldsymbol{\beta vs.\alpha}$ | 0.33 | 0.019 | 17.40 | <2e-16 |
| **Trial** | -0.018 | 0.0001 | -17.61 | <2e-16 |
| **Type x Trial** | -0.019 | 0.001 | -16.10 | <2e-16 |
| **Group** $\left[ {D_{KL}(\alpha}_{par}^{m} \right)]$ | 0.120 | 0.040 | 2.94 | 0.004 |
| **Group** $\left[ {D_{KL}(\beta}_{par}^{m} \right)]$ | -0.090 | 0.040 | -2.17 | 0.032 |
