# Supplementary material for: Self-other generalisation shapes social interaction and is disrupted in borderline personality disorder
Source: eLife. 2025 Jul 14;14:RP104008. doi: 10.7554/eLife.104008 (PMC12259023; doi:10.7554/eLife.104008)
Supplement: Supplementary file 4. — Bootstrapped results with their 95% CI with and without group status regressed against psychometric variables. [file elife-104008-supp4.docx]

| Original | | | | | |
| --- | --- | --- | --- | --- | --- |
| **Node 1** | **Node 2** | **mean** | **sd** | **lower** | **upper** |
| CTQ | MZQ | 0.40 | 0.08 | 0.23 | 0.56 |
| MZQ | RGPTSB | 0.39 | 0.08 | 0.23 | 0.54 |
| CTQ | $\vert\Delta\alpha_{ppt}^{m}\vert$ | -0.25 | 0.10 | -0.46 | -0.04 |
| MZQ | $\vert\Delta\beta_{ppt}^{m}\vert$ | -0.25 | 0.09 | -0.44 | -0.07 |
| Group Status Controlled | | | | | |
| **Node 1** | **Node 2** | **mean** | **sd** | **lower** | **upper** |
| CTQ | MZQ | 0.18 | 0.10 | 0.004 | 0.35 |
| MZQ | RGPTSB | 0.32 | 0.09 | 0.13 | 0.50 |
| CTQ | $\vert\Delta\alpha_{ppt}^{m}\vert$ | -0.19 | 0.09 | -0.380 | -0.01 |
| MZQ | $\vert\Delta\beta_{ppt}^{m}\vert$ | -0.13 | 0.11 | -0.35 | 0.09 |
