# Supplementary material for: Self-other generalisation shapes social interaction and is disrupted in borderline personality disorder
Source: eLife. 2025 Jul 14;14:RP104008. doi: 10.7554/eLife.104008 (PMC12259023; doi:10.7554/eLife.104008)
Supplement: Supplementary file 5. [file elife-104008-supp5.docx]

**Reaction times in the Intentions Game**

*Phase 1*

Examining reaction times (in milliseconds; ms) in phase 1 by choice type revealed that, compared to competitive choices, individualistic choices were made faster (linear estimate = -880.60, 95%CI: -1385.42, -376.2; t = -3.42, p < 0.001), and prosocial choices were made fastest (linear estimate = -1171.1, 95%CI: -1701.97, -640.71; t = -4.32, p < 0.001) irrespective of the type of choice pair. Prosocial choices were made significantly faster than individualistic choices (linear estimate = -290.70, 95%CI: -548.50, -32.91; t = -2.21, p = 0.027).

*Phase 2*

All participants were slower at the start of phase 2 and sped up over time (linear estimate = -15.03, 95%CI: -21.06, -8.99; t = -4.88, p < 0.001). Baseline participant-partner similarity did not have an overall effect on reaction time but did interact with trial – as participant-partner similarity increased, reaction times early in phase 2 were significantly slower and this effect attenuated over time (linear estimate = -0.53, 95%CI: -0.75, -0.32; t = -4.91, p < 0.001; see **Figure 3-figure supplement 4**). Reaction time did not vary between groups: both BPD and CON participants displayed the same effect.

Reaction times and belief updates in phase 2 were significantly coupled, such that larger shifts in posterior beliefs along both axes were associated with larger reaction times (linear estimate$\left[ {D_{KL}(\alpha}_{par}^{m} \right)]$ = 0.044, 95%CI: 0.027, 0.06, t = 5.01, p < 0.001; linear estimate$\left[ {D_{KL}(\beta}_{par}^{m} \right)]$ = 0.021, 95%CI: 0.005, 0.039, t = 2.49, p = 0.012).

*Phase 3*

Reaction times in phase 3 revealed that compared to competitive choices, individualistic choices were made faster (linear estimate = -528.50, 95%CI: -943.60, -114.6; t=-2.50, p = 0.012), and prosocial choices were made fastest (linear estimate = -693.5, 95%CI: -1137.65, -250.39; t=-3.07, p = 0.002). Prosocial choices were no longer executed significantly faster than individualistic choices. All participants made faster choices in phase 3 compared to phase 1 (linear estimate = -242.02, 95% CI: -332.64, -151.41; t=-5.24, p = 0.001).
